# Sunscreen Enhancement of Octyl Methoxycinnamate Microcapsules by Using Two Biopolymers as Wall Materials

**DOI:** 10.3390/polym13060866

**Published:** 2021-03-11

**Authors:** Chuntao Xu, Xuemin Zeng, Zujin Yang, Hongbing Ji

**Affiliations:** 1 School of Chemistry and Chemical Engineering, Guangxi University, Nanning 530004, China; chuntao@zspt.edu.cn; 2 School of Information Engineering, Zhongshan Polytechnic, Zhongshan 528400, China; 3 Fine Chemical Industry Research Institute, School of Chemistry, Sun Yat-Sen University, Guangzhou 510275, China; zengxm9@mail2.sysu.edu.cn; 4 School of Chemical Engineering and Technology, Sun Yat-Sen University, Zhuhai 519082, China; 5 School of Chemical Engineering, Guangdong University of Petrochemical Technology, Maoming 525000, China

**Keywords:** octyl methoxycinnamate, sunscreen, microcapsule, complex coacervation, synergistic effects

## Abstract

Octyl methoxycinnamate (OMC) is widely used as a chemical sunscreen in sunscreen cosmetics. However, its direct contact with the skin would bring certain risks, such as skin photosensitive reaction. How to improve the effect of skin photodamage protection has become a current research hotspot. Encapsulating ultraviolet (UV) filters into microcapsules is an interesting method to increase the photostability of filters. In this study, sodium caseinate (SC) and arabic gum (GA) are chosen as wall materials to prepare synergistic sunscreen microcapsules by complex coacervation technology. A series of experiments are conducted to investigate the effects of pH, wall material concentration, and wall/core ratio on the formation of OMC microcapsules. The morphology, composition, and stability of OMC microcapsules are characterized by scanning electron microscopy (SEM), Fourier transform infrared spectroscopy (FTIR), and thermogravimetric analysis (TGA). The OMC microcapsule is uniform in size distribution, smooth in surface morphology, and has good thermal stability. The results show that the ultraviolet absorption of the OMC microcapsules is better than that of the uncoated OMC for the ultraviolet-B (280–320 nm). Moreover, the OMC microcapsule released 40% in 12 h, while OMC released 65%, but the sun protection factor (SPF) of the OMC microcapsule sunscreen is 18.75% higher than that of OMC. This phenomenon may be attributed to the hydrophobic interaction between SC and OMC and the electrostatic interaction between SC and GA.

## 1. Introduction

In recent decades, the destruction of the atmospheric ozone layer has made people suffer from excess exposure of ultraviolet (UV) radiation, causing serious damage to the human health [1,2]. It has been reported that about 1.5 million disability-adjusted life years (DALYS) in the world were related to UV radiation [3]. Based on the wavelength and energy, UV radiations are mainly divided into three bands: short-wave ultraviolet C (UVC, 100–280 nm), medium-wave ultraviolet B (UVB, 280–320 nm), and long-wave ultraviolet A (UVA, 320–400 nm), respectively [4,5]. UVC with the weakest penetration force could be completely absorbed by the atmosphere, but 5–10% of UVB and 90–95% of UVA radiation could penetrate the clouds and the ozone layer to the earth surface, causing harm to our skin [6,7]. For example, UVB overexposure to skin would lead to inflammation, immunosuppression, and skin cancer [8]. Therefore, it is particularly important to effectively avoid UVB absorption in life. Some methods have been used to protect our skin including physical or chemical filters or absorbing and reflecting UV radiation [9]. Sunscreen products are the main skin protection against the damage of UVB.

Currently, octyl methoxycinnamate (OMC) is one of the most common sunscreen agents in commercially available cosmetics in the market to resist UVB in sunlight owing to its excellent UV absorption curve, high lipophilicity, and good oil solubility [10,11]. However, during its use, there has been some disadvantages such as poor photostability and strong permeability [12]. According to previous reports, the large amount of OMC would accumulate in the deeper layers of skin after coating on the skin superficial areas. OMC may inhibit the deiodinase activity, induce morphological changes in the testes and ovaries of rats [13,14,15], and affect secretion of estrogen, reproductive development, and nervous system of rat offspring [16]. Recently, efforts have been made to develop some more efficient and safe sunscreen agents, reducing the toxicological risk from the percutaneous absorption. In order to achieve this goal, many strategies have been employed to prevent photodamage to skin [17]. Microencapsulation is a new inclusion method where active materials are encapsulated in the microcapsules. By changing the wall materials, the microcapsules can effectively protect and control release of the enclosed active ingredients [18,19,20,21]. It was a promising technology to encapsulate OMC for overcoming the trans-epidermal penetration and promoting UV absorption ability. Complex coacervation, as one kind of microencapsulation technology, is accomplished by phase separation of one or many hydrocolloids from the initial solution and the subsequent deposition of the newly formed coacervate phase around the active ingredient which has been used widely to microencapsulate the active components [22,23,24,25].

**Scheme 1:**
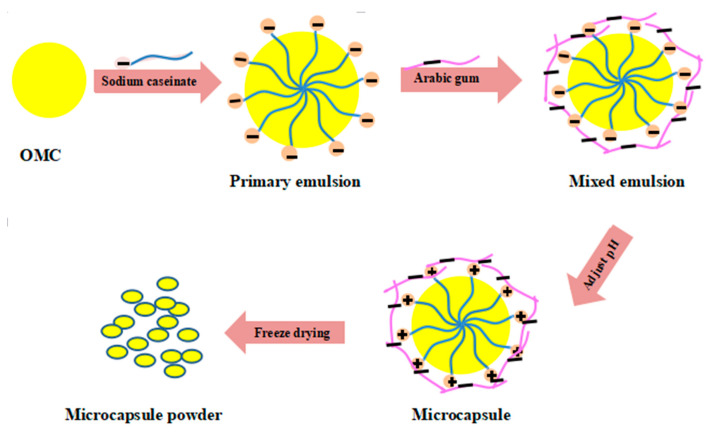
Preparation of octyl methoxycinnamate (OMC) microcapsule by complex coacervation.

In the present study, sodium caseinate (SC) and arabic gum (GA) are chosen as wall material to prepare OMC microcapsules based on the complex coacervation method (Scheme 1). The process conditions of the microcapsules were optimized by adjusting pH, wall material concentration, and wall/core ratio. Scanning electron microscopy (SEM), Fourier transform infrared spectroscopy (FTIR), and thermogravimetric analysis (TGA) are used to characterize the morphology, structures, and stability of the OMC microcapsules. Finally, the encapsulation efficiency, release, and sun protection performance of the OMC microcapsules are also investigated.

## 2. Materials and Methods

### 2.1. Materials and Reagents

The sunscreen octyl methoxycinnamate (OMC) was purchased from Shanghai Aladdin Bio-Chemistry Technology Co., Ltd. (Shanghai, China). Sodium caseinate (SC) was supplied by Shanghai Jiecong Trading Co., Ltd. (Shanghai, China). Arabic gum (GA) was obtained from Guangzhou Zhuoxin Bio-s Co., Ltd. (Guangzhou, China). Glacial acetic acid and glutaraldehyde were provided by Shenzhen Yinuo Food Ingredients Co., Ltd. (Shenzhen, China) and Guangzhou Mingwang Biotechnology Co., Ltd. (Guangzhou, China), respectively. Other chemicals and solvents used in this work were of analytical grade and purified by general methods.

### 2.2. Selection of Wall Materials

In order to obtain sunscreen-loaded microcapsules with good sunscreen effect, it was necessary to study the OMC microcapsules by using the different wall materials with the good ultraviolet absorption. Several kinds of wall materials, such as modified starch (C001), sodium carboxymethyl cellulose (CMC), arabic gum (GA), sodium alginate (ALG), chitosan (CS), sodium caseinate (SC), maltodextrin (MD), and β-cyclodextrin (β-CD), were measured by UV spectrophotometer (LC-16, Shimadzu, Kyoto, Japan) during 200–400 nm.

### 2.3. Preparation of OMC Microcapsules

In this study, the complex coacervation method was used to prepare OMC microcapsules as follows: a series of SC or GA solutions (1.0%) were obtained by dissolved SC or GA in the buffered solutions with different pH values (2.5, 3.0, 3.5, 4.0, 4.5, and 5.0). Then, the zeta potentials of solutions were measured by dynamic light scattering (DLS, Malvern Instruments, Worcestershire, UK). According to the values of the zeta potentials, the theoretical strength of electrostatic interactions (*SEI*) was calculated as:

(1) $$SEI=\left|Z_{1}\right|\times \left|Z_{2}\right|$$

where *Z*_1_ and *Z*_2_ represent the zeta potential of SC and GA, respectively.

Dissolving 0.3 g OMC in 15 mL SC solutions (1.0%, *w*/*w*). After homogenizing at 50 °C for 5 min, the mixture was added into a series of 1.0% GA solutions (1.5, 3.0, 6.0, 8.0, 12.0, 15.0, 18.0, and 24.0 mL). After stirring at 500 rpm for 2 h (RW20, IKA, Shanghai, China), glacial acetic acid was added to the above solution dropwise to adjust the pH of solutions to a certain value (2.5, 3.0, 3.5, and 4.0). The obtained solutions were stirred at 500 rpm for 2 h at room temperature. The samples were dried under vacuum with a freeze dryer (Martin Christ GmbH, Osterode am Harz, Germany) to obtain OMC microcapsules. The amount of OMC in the OMC microcapsules was determined by high-performance liquid chromatography at λ = 310 nm (HPLC, LC-16, Agilent, Santa Clara, CA, USA)), detected at a wavelength of 310 nm, and calculated by drawing the standard curve of OMC. Each experiment was performed in triplicate and standard deviation was used as the error bars.

(2) $$LC\;\left(\%\right)=\frac{\mathrm{The}\;\mathrm{amount}\;\mathrm{of}\;\mathrm{OMC}\;\mathrm{in}\;\mathrm{the}\;\mathrm{microcapsules}}{\mathrm{The}\;\mathrm{total}\;\mathrm{microcapsules}}\times 100$$

(3) $$EE\;\left(\%\right)=\frac{\mathrm{The}\;\mathrm{amount}\;\mathrm{of}\;\mathrm{OMC}\;\mathrm{in}\;\mathrm{the}\;\mathrm{microcapsules}}{\mathrm{The}\;\mathrm{total}\;\mathrm{OMC}}\times 100$$

### 2.4. Experimental Designs of OMC Microcapsules

The factors that may affect encapsulation efficiency of OMC microcapsules were studied mostly by using an orthogonal experiment, and they included stirring speed, ratio of wall material to core material, and content of curing agent. Then, the factors that influence the characteristics of hydrothermal products were selected on the basis of the same experiment to conduct the single-factor test. The current study mainly identified encapsulation efficiency of OMC microcapsules at stirring speed ranging between 200 and 400 rpm, with a ratio of wall material to core material ranging from 1:2 to 2:1 and content of curing agent of 1–3%. The experimental conditions are displayed in Table 1.

### 2.5. Characterization of OMC Microcapsules

The mean size, size distribution, and the zeta potential of the OMC microcapsules were recorded by DLS with a Zetasizer Nano ZS (Malvern Instruments, Worcestershire, UK). The average diameters and size distributions were acquired by measuring 100 droplets individually (Image J software, National Institutes of Health, Bethesda, MD, USA), for which the model is expressed as:

(4) $$D_{nL}=\frac{\sum^{\;}nd}{\sum^{\;}n}$$

The morphology of OMC microcapsules was observed by a Hitachi S-520 scanning electron microscope (SEM, Hitachi, Tokyo, Japan) with accelerating voltage of 15 kV. FTIR spectra (Bruker TENSOR II, Karlsruhe, Germany) were used to analyze the structure of the test samples in the wavelength range of 4000–400 cm^−1^ at 4.0 cm^−1^ resolution. The thermal stability of OMC, SC, and GA complex and OMC microcapsules was determined by using a NETZSCH STA 449C thermal analysis system (Selb, Germany).

### 2.6. Release of OMC from the OMC Microcapsules

The release of OMC from the microcapsules was also investigated using the dialysis method [26,27], with some modification. Briefly, 10 mg of OMC microcapsules was dissolved in 5 mL of phosphate-buffered solution (PBS) at pH = 6.5 under ultrasonic irradiation (KQ-400DE, Kunshan, China). 1 mL of solution was sampled and diluted to 4 mL with PBS, and dialyzed with 30 mL of the PBS by using a dialysis tube (molecular weight cut-off (MWCO): 3500 Da) at 37 °C. At predetermined time intervals, 0.5 mL of release media was withdrawn and the same volume of fresh PBS was added. The amount of released OMC was determined by HPLC (λ = 310 nm). For comparison, the release of free OMC without SC and GA complex was performed in the same condition [22]. The cumulative releases were calculated as,

(5) $$\mathrm{Release}(\%)=\frac{(V_{e}\sum_{0}^{n-1}C_{i}+V_{0}C_{n})\times 100}{m}$$

where *V*_0_, *C_n_*, *V_e_*, *C_i_*, and *m* represented the total volume of outside solutions, the concentration of OMC at time *n*, the volume taken out each time, the concentration of OMC at time of *i*, and the total mass of OMC at the beginning, respectively.

Mathematical model of OMC release is an efficient method to understand the delivery of bioactive molecules, which can understand factors affecting the release rate and reduce the number of experiments. The release behavior of the OMC microcapsule was investigated by four kinds of kinetics mode, including zero order, first order, Higuchi, and Rigter-Peppas model to obtain the best kinetics mode based on the highest *R^2^*,

(6) $$\frac{R_{t}}{R_{\infty }}=kt$$

(7) $$\frac{R_{t}}{R_{\infty }}=1-e^{-kt}$$

(8) $$\frac{R_{t}}{R_{\infty }}=kt^{1/2}$$

(9) $$\frac{R_{t}}{R_{\infty }}=kt^{n}$$

where *R_t_* is the release amounts of OMC at *t*, *R*_∞_ is the total release amounts of OMC at infinite time, *R_t_/R*_∞_ is the fraction of OMC released at *t*, and *k* is a constant, respectively. Exponent *n* is usually used to characterize four types of transport of active molecules through the matrix [7], which describes the kinetic and mechanism of OMC release. When *n* ≤ 0.43, the major driving force is the Fickian diffusion, if *n* is in the range of 0.43 and 0.85, the release mechanism is the diffusion and the swelling and *n* = 0.85 indicates zero order release kinetics, and *n* > 0.85 demonstrates Super Case-II transport [24].

### 2.7. UV Absorption Effect of the OMC Microcapsules

According to “The evaluation method of sunscreen effect of sunscreen cosmetic-UV absorbance method”, the sunscreen performance of the OMC microcapsules was tested by an UV/vis near-infrared spectrophotometer (UV-3600 Plus, Shimadzu, Kyoto, Japan), and its wavelength was from 200 to 800 nm. In detail, the same content of sunscreen and the OMC microcapsules were smeared on the 3 M tape and further scanned from 280 to 400 nm. Finally, the UV absorption map was drawn.

### 2.8. Test of Sun Protection Factor (SPF) Value

The OMC microcapsules were used to test its sunscreen ability and sustained-release performance according to the reported method [25]. The sunscreen formulations were prepared according to the 2015 edition “cosmetic safety technical guide” (Table 2), as follows: phase A and phase B are heated to 75–80 °C respectively, and stirred continuously until all the components are dissolved, and until the liquid becomes clear when necessary. Phase C is added to the mixture of phase A and phase B, heated to 75–80 °C, stirred homogeneously with high shear dispersion emulsifier (RW20, IKA, Shanghai, China), and then neutralized with lactic acid or sodium hydroxide. Finally, the mixture was cooled to room temperature, and used to test the SPF value of sunscreen products in vitro. Four samples (sunscreen of OMC, sunscreen of OMC microcapsules, mixed sunscreen, and sunscreen from market) of SPF value were tested by SPF-290AS (Solarlight, Glenside, PA, USA) in vitro. Each experiment was performed in triplicate and standard deviation was used as the error bars.

### 2.9. Statistical Analysis

The results were evaluated according to the analysis of variance (ANOVA), and the means were compared by the Tukey test, considering the significance level of 5% (*p* < 0.05), using Statistica 7 software (StatSoft, Tulsa, OK, USA).

## 3. Results and Discussion

### 3.1. Screening of Natural Polymer Wall Materials

**Figure 1:**
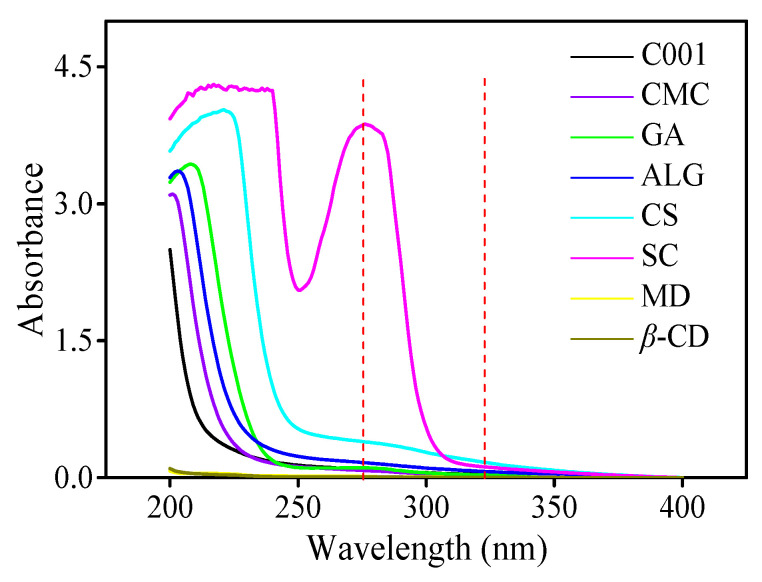
Ultraviolet (UV) absorption effect of wall material.

As shown in Figure 1, the absorbance of the different wall materials was different in UVB (280–320 nm), and the order of UV absorption was SC > CS > ALG > GA > C001 = CMC > MD = β-CD. In this region (280–320 nm), SC has the highest UV absorbance. It is attributed that SC is composed of α-, β-, γ-, and *κ*-casein, which contain a large number of aromatic amino acids such as tyrosine and tryptophan, which have a relatively large conjugated structure in the molecule with a strong absorption peak around 280 nm. Therefore, it has been used as one of the wall materials of sunscreen microcapsules. CS also has good UV absorption, because CS is mainly composed of amino-d-glucose and *N*-acetyl-d-glucosamine, with a certain number of double bonds in the molecule and relatively high absorbance. However, CS is an acid-soluble substance (pH < 6) and can dissolve in the buffer solution (pH < 5). This pH is close to the isoelectric point of SC, and the pH range of the two for complex coagulation is too small, so it is not suitable for the wall material for the complex coagulation experiment. The ultraviolet absorption of ALG and GA is similar. Compared with ALG, GA has better solubility and lower viscosity, which is beneficial to the fluidity and dispersity of the capsule. Through comprehensive comparison, SC and GA were selected as wall materials to prepare sunscreen microcapsules by complex coacervation.

### 3.2. Preparation of the OMC Microcapsules

**Figure 2:**
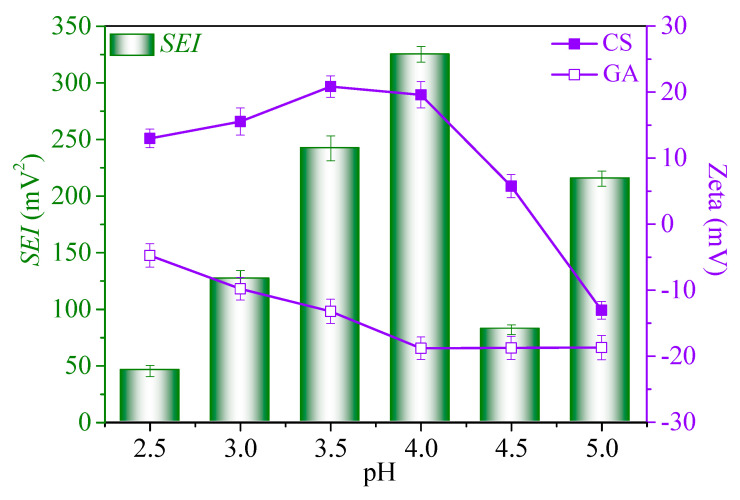
The zeta potentials (mV) and *SEI* (mV^2^) measurement of the GA and SC in different pH values. Means of values obtained in triplicate (*p* ≤ 0.05).

The zeta potentials of GA and SC at different pH are studied by DLS. As shown in Figure 2, GA solutions showed zeta potentials, and the negative charges increased with increasing the pH from 2.5 to 5, which were due to the ionization of carboxyl groups in GA in acid environment. SC solutions showed positive zeta potentials when pH < 4.5, were close to 0 when pH was 4.5, but they were negative when pH > 4.5. The significant pH changes in SC solution were due to the presence of some carboxyl and amine groups in SC. When pH < 4.5, SC solution showed positive zeta potentials due to the protonated amine groups, and negative zeta potentials when pH > 4.5, which was ascribed to the carboxyl group ionization, and the zeta potential of SC was close to zero when the equal groups of amine and carboxyl protonated or ionization were at isoelectric point [26]. As shown in Figure 2, the maximum *SEI* value appeared at pH = 4.0, implying the strongest electrostatic interaction between SC and GA. However, the lowest *SEI* value was observed when pH of the solutions of SC and GA was at 2.5.

**Figure 3:**
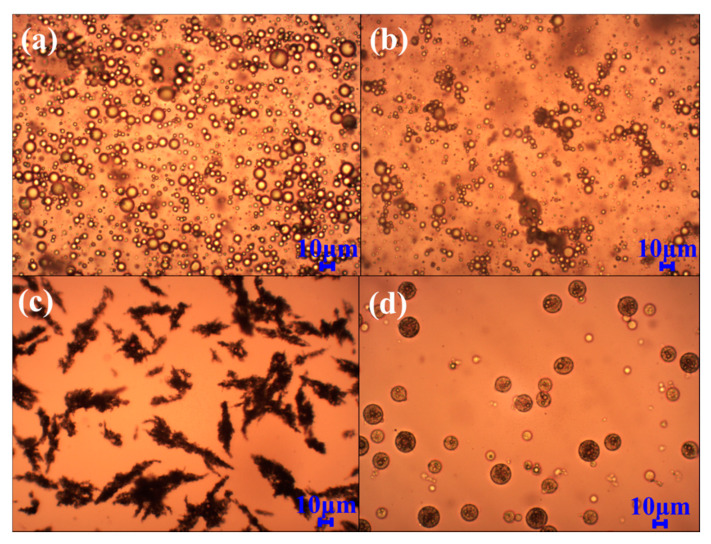
The picture of SC/GA by complex coacervation at different pH. (**a**) pH = 4.0, (**b**) pH = 3.5, (**c**) pH = 3.0, (**d**) pH = 2.5.

The OMC microcapsules were prepared by the complex coacervation method with SC and GA as wall materials and OMC as core materials at different pH. Figure 3 shows the morphology of the OMC microcapsules by metalloscope. Figure 3a shows many oil droplets with different particle sizes, suggesting the failed preparation of the OMC microcapsules at pH = 4.0. It could be seen from Figure 3b that OMC was encapsulated by SC and GA as wall materials with dendritic shape, suggesting that some OMC were included in the core of GA/SC wall materials at pH = 3.5. From Figure 3c, OMC was encapsulated by SC and GA with dendritic shape, implying the preparation of the OMC microcapsules at pH = 3.0. As shown in Figure 3d, the spherical microcapsules were observed with core-shell structure, indicating that the OMC microcapsules were prepared successfully at pH = 2.5. The phenomenon was mainly due to the electrostatic interaction between SC and GA [27]. The higher pH (2.5–4.0) was not conducive to the formation of the OMC microcapsules due to the stronger electrostatic interaction between SC and GA. The optimized pH of the OMC microcapsules with different ratios of SC and GA as wall materials was 2.5 in this study.

**Figure 4:**
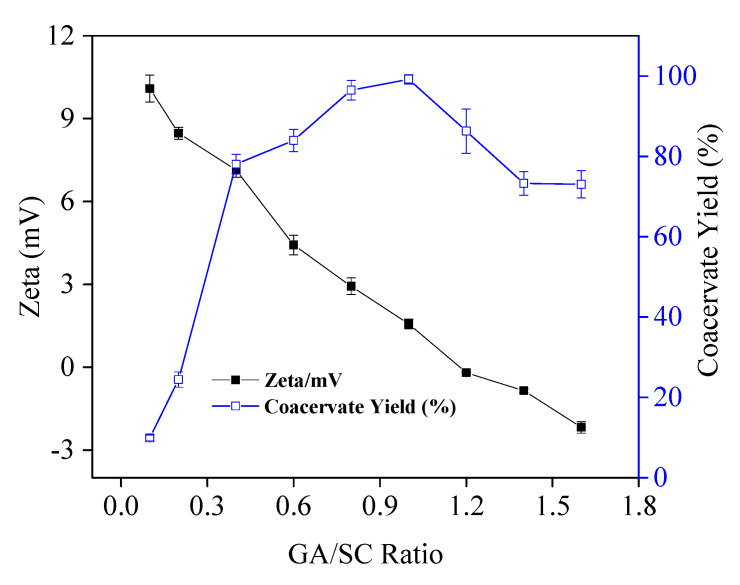
Coacervation of SC and GA as a function of mixing ratio. Zeta potential of the OMC microcapsules were recorded by dynamic light scattering (DLS) with a Zetasizer Nano ZS (Malvern Instruments, Worcestershire, UK). Means of values obtained in triplicate (*p* < 0.05).

Ratio of mixture of GA/SC is important for the charge balance and the intensity, and the degree of self-aggregation during complexation. Ratio of SC and GA on the coacervation were further studied and shown in Figure 4 and Table 3. The zeta potentials were observed to change from positive values to negative values with increasing the content of GA. The zeta potential was close to zero when the ratio of GA and SC was 1:1, indicating that the GA and SC were completely neutralized by each other. The further increased ratio of GA and SC could make the OMC microcapsules solutions have negative zeta potentials [28]. In addition, the greatest coacervate yield was observed at the GA/SC ratio 1:1, and a further increase or decrease of the ratio can decrease the coacervation significantly. Therefore, the optimized ratio of GA and SC was 1:1. When the wall material ratio is 1:1, the mean particle size is 10.01 ± 0.012 μm and the polydispersity index (PDI) is 0.202 ± 0.002. The distribution of capsules was uniform. It was also found by Thaiane et al. [29] that the zeta potential of gelatin and arabic gum and the ratio of 1:1 were determined as the most suitable for microencapsulation, as the best condition for the microcapsule formation.

**Figure 5:**
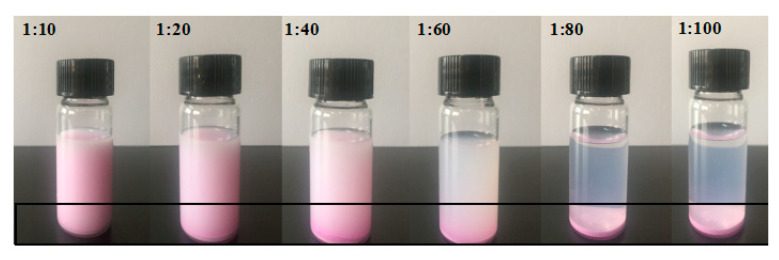
Effect of ratio of emulsifier/core materials on the formation of the OMC microcapsules.

Emulsion stability is important for the formation of microencapsulation, which was affected by the ratio of emulsifier/core materials, and further influenced the characteristics of the OMC microcapsules. The effect of the ratio of OMC and GA/SC on the preparation of the OMC microcapsules was investigated and shown in Figure 5. From Figure 5, it can be seen that a gradual phase separation occurred, and the pink emulsions were gradually divided into two layers of supernatant and pink precipitation with the decrease of the emulsifier. This phenomenon was mainly caused because: (1) the OMC-loaded micelles may be forced based on the high concentration of emulsifier and dispersed in the solutions, and OMC could not be included in GA/SC complex, (2) the contact between SC and OMC increased with the decrease of emulsifier concentration, and the complex condensation and settlement of the core material would gradually occur. The content of OMC in the solution was decreased gradually, and the solution became clear from turbidity to clarification. (3) OMC was completely emulsified by GA/SC at the low concentration of emulsifier, leading to the obtained OMC microcapsule by complex coacervation settled at the bottom, and no OMC appeared in the solutions.

**Figure 6:**
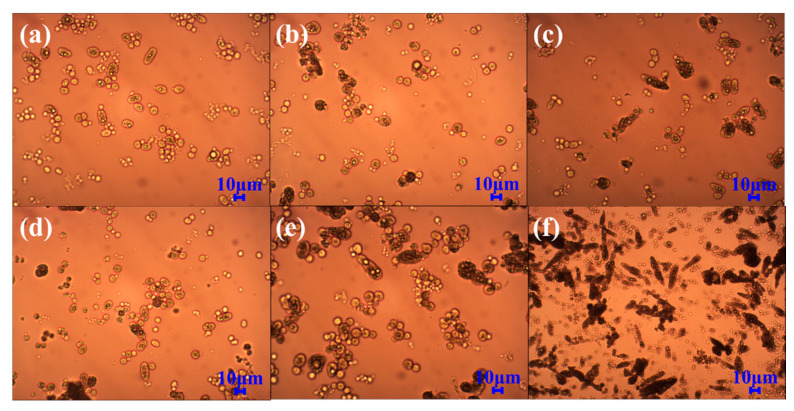
Effect of wall material concentration on the formation of the OMC microcapsules, recorded by DLS with a Zetasizer Nano ZS with ×400. (**a**) 1%, (**b**) 1.2%, (**c**) 1.4%, (**d**) 1.6%, (**e**) 1.8%, (**f**) 2.0%.

Wall material is usually used to include the core material in its structure during processing and storage. However, high wall material load often resulted in poor retention. In this study, the effects of the concentration of wall material in solution on the preparation of the OMC microcapsules was also investigated and shown in Figure 6. From Figure 6, it can be seen that the concentration of GA/SC showed a significant effect on the formation of the OMC microcapsules, in which the concentration of 1.0% offered the spherical shape with the uniform size. However, we would not make SC disperse in GA solutions with the increase in the concentration of SC/GA, changing charge during the process of adjusting pH [30]. Too much charge may lead to an intense reaction, resulting in the appearance of irregular microcapsules. Therefore, the concentration of SC/GA for the optimization of the preparation process of sunscreen-loaded microcapsules was 1.0%, indicating the important interaction between OMC and SC/GA.

The optimization of the preparation process of the OMC microcapsules was further studied by an orthogonal experiment (Table 4). The influence of curing rate, OMC to GA/SC ratio, and curing agent content on the encapsulation efficiency (*EE*) of the OMC microcapsules was studied. The results showed that the values of *R* are in the order of curing agent content (0.670) > curing rate (0.135) > wall-core ratio (0.093), indicating that curling agent content is the most important factor, followed by stirring speed and core material ratio. The results also showed that the lower *EE* would be found at higher content of curing agent, due to the reversible reaction for the complex coacervation. Therefore, the optimized preparation process was as follows, 200 rpm, ratio of 1:1 of OMC to GA/SC, and 1% curing agent content. The *EE* and *LC* of the OMC microcapsules were 83.3% and 41.7%, respectively.

### 3.3. Characterization of the OMC Microcapsules

**Figure 7:**
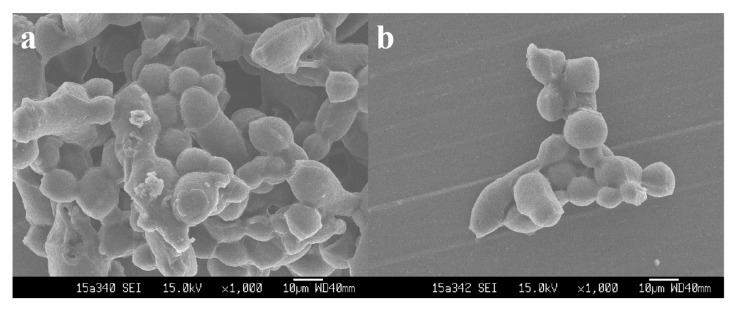
Scanning electron microscopy (SEM) of the microencapsulated OMC. (**a**) Structure of microcapsule aggregates, (**b**) structure of several microcapsules connected together.

The formation of the OMC microcapsules prepared by the optimized process was confirmed by SEM observation. The outer morphology of the microcapsules is presented in Figure 7. It can be seen that the microcapsules are bonded to each other, which was common in the freeze-drying process by matrix contract during the drying process [29]. The complete microcapsules can ensure the effective protection of the encapsulated OMC (Figure 7a). The microcapsules had spherical morphologies with smooth surface appearance, which were micro-sized, and the average diameter was about 10 μm based on 100 particles (Figure 7b). The result of microcapsules was similar to the results reported by Michele et al. [31], and the results are consistent with the results of the metalloscope.

**Figure 8:**
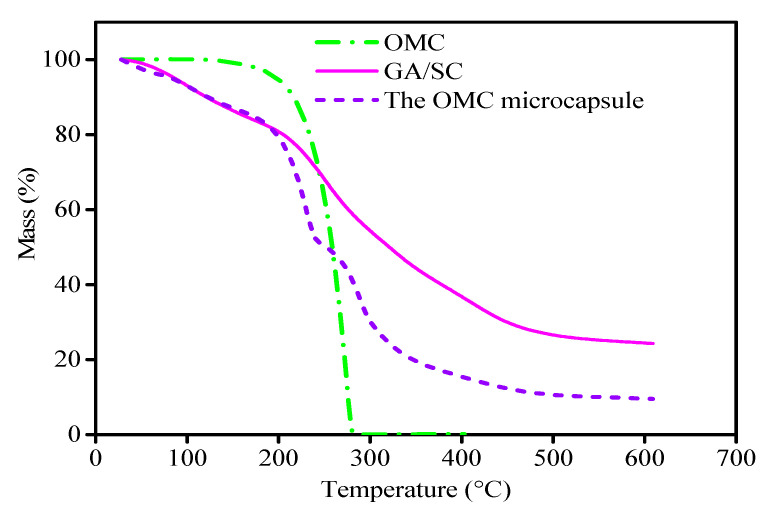
Thermal behavior of OMC, SC/GA, and the OMC microcapsule.

The thermal stability of OMC and GA/SC as wall material and the OMC microcapsules was investigated by thermogravimetric analysis, as shown in Figure 8. The thermogram for the pure OMC showed one thermal event, at 200–280 °C, which was due to the volatilization of OMC [32]. The GA/SA had three thermal degradation events, the first between 25 °C and 150 °C, corresponding to weight loss of the remaining water in the sample with a mass loss of about 15%. Another weight lost was observed from 211.8 °C to 470 °C, the temperature range where GA or SA degradation occurs, corresponding to 60% of weight loss. The third weight loss was due to the carbonization process, with 10% of weight loss. The thermal degradation process of the OMC microcapsule presented four thermal events. The first was in the range from 25 to 150 °C, with 15% mass loss due to evaporation of water. In the second event (220–300 °C), there was a significant mass loss, about 70%, indicating the release of the core material. In the third event (320–420 °C), there was a mass loss of about 10%, which was due to the decomposition of GA/SC, and the GA/SC used as wall materials may have caused this high weight loss [33]. The fourth weight loss was due to the carbonization process, with 8% of weight loss. The results showed that the OMC thermal stability was significantly improved by the encapsulation process [34]. This increased thermal stability can be due to the thermal resistance of GA/SA used as a microcapsule casing, resulting in a slower release.

**Figure 9:**
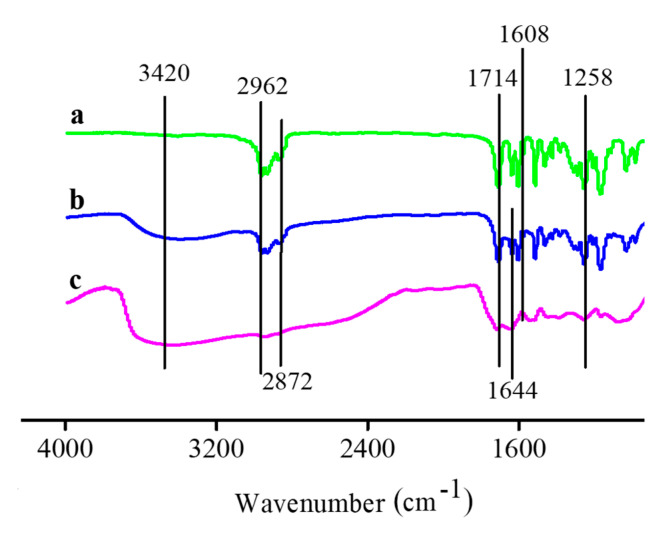
Fourier transform infrared (FTIR) spectra of OMC (**a**), OMC microcapsules (**b**), and the SC/GA (**c**).

FTIR was applied to estimate the interactions of the OMC and SC/GA using OMC and SC/GA as control. FTIR spectra of OMC, GA/SC, and the OMC microcapsules are shown in Figure 9 in the range of 4000–500 cm^−1^. The spectra of all samples, except for free OMC, showed a stretching band at 3420 cm^−1^ corresponding to –OH groups [9]. For OMC, the strong peaks around 2962 and 2872 cm^−1^ are due to the C–H stretching modes of CH_2_ and CH_3_. In addition, the absorption band at 1714 cm^−1^ was ascribed to C=O stretching vibration. At 1608 cm^−1^, a vibration band was assigned to the deformation of C=C originated from an alkene group, also present in the molecular structure of this compound. In the same spectrum, other typical bands of OMC were observed at 1466 cm^−1^ assigned to C=C group characteristics of the aromatic ring; at 1258 cm^−1^, asymmetric deformation of C–O–C, and at 984 cm^−1^, assigned to vibrations bands of C–H bond characteristics of the aromatic ring [11] (Figure 9a). FTIR spectra of SC/GA displayed a strong peak at 3420 cm^−1^, which was due to the O–H and stretching vibration, and no obvious bands appeared at 2962 and 1714 cm^−1^, respectively [11] (Figure 9b). However, the strong peaks at 3420, 2962, and 1714 cm^−1^ were observed in the FTIR spectra of the OMC microcapsule, indicating that OMC was successfully entrapped by SC/GA as wall material and no interaction occurred between OMC and wall material (Figure 9c). This result was in accordance with previous results showing that OMC were bound to SC/GA via H-bonding [13].

### 3.4. Release Behavior of the OMC Microcapsules

**Figure 10:**
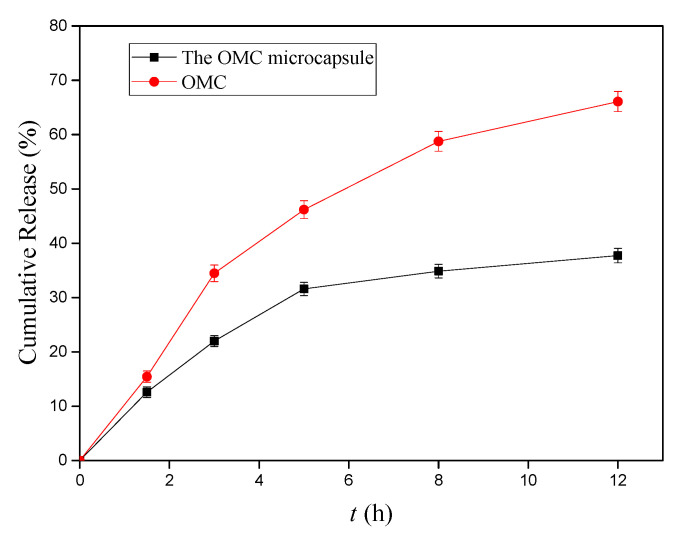
Cumulative release of OMC and the OMC microcapsule.

The release property of OMC was a key factor for the idea sunscreen-loaded microcapsule. As shown in Figure 10, prepared under optimal conditions, free OMC showed a fast release rate at the beginning and then tended to equilibrium. After 12 h, OMC microcapsules released about 40% of OMC, while free OMC released more than 65%. The release rate of OMC was about 30% higher than that of the OMC microcapsule. The phenomenon could be explained as: (1) the microcapsules could protect the OMC from fast release, and (2) SC/GA have good film-forming properties, which made OMC-loaded microcapsules dense with the smooth surface and could not dissolve in aqueous solution after curing, leading to effectively inhibit the release of OMC. These results show that the OMC-loaded microcapsule could effectively control the release of OMC, implying that strong interactions may exist between OMC and GA/SC [7,23].

In order to deeply reveal the release mechanism of OMC microcapsules, various mathematical models, such as zero-order kinetics model, first-order kinetics model, Higuchi model, and Korsmeyer-Peppas model, were applied to fit the experimental data, using the free OMC as control [23]. As shown in Table 5, the release behavior of the OMC microcapsule conformed to the Rigter-Peppas model because of the higher *R*^2^ values (0.99). For the Rigter-Peppas model, *n* gave an indication of the release mechanism [35]. The *n* values in the Rigter-Peppas equation were in the range of 0.43 and 0.85 (Table 5), implying that the diffusion and relaxation rates were the dominant mass transfer mechanism. Thus, the release of OMC from the spherical-shaped microcapsules is driven by a non-Fickian diffusion or anomalous diffusion release phenomenon [30].

### 3.5. UV Absorption Effect of the OMC Microcapsules

**Figure 11:**
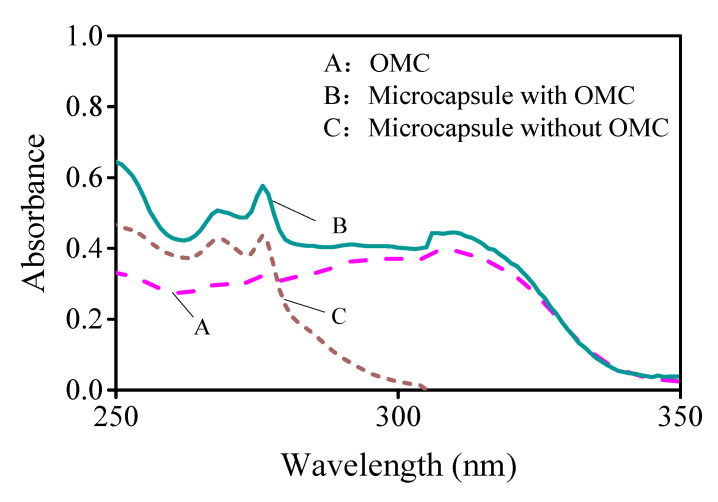
Ultraviolet absorption effect of the OMC, OMC microcapsule, and microcapsule without OMC.

A good sunscreen formulation is very important, which not only depends on the physicochemical properties of the filters, but also on the OMC microcapsule’s delivering capacity [35]. In this study, the sunscreen effect of the OMC microcapsule was studied by using a UV spectrophotometer. As shown in Figure 11, the free OMC and SC had an obvious absorption peak between 280 and 320 nm, but the OMC microcapsule also had the same absorbance in the range of 320–400 nm, indicating that the microcapsule consisted of SC and OMC [36]. The maximum absorbance of the OMC microcapsule was larger than that of the free OMC, implying that the sunscreen effect of the OMC microcapsule might be better than that of free OMC (Figure 11). It was attributed that the OMC microcapsule had the UV absorption function of chemical sunscreen, and also had the effect of physical sunscreen on ultraviolet reflection. In detail, the OMC encapsulated by the microcapsule still had a good effect of sunscreen, the wall material also had the UV absorption, and the OMC microcapsule as a spherical particle with micro-size had different degrees of scattering and refraction to ultraviolet rays and absorbing ultraviolet rays [5]. The enhanced sunscreen effect was due to the synergistic effect of OMC and GA/SC [15].

### 3.6. Effect of Microencapsulation on SPF of Sunscreen

**Figure 12:**
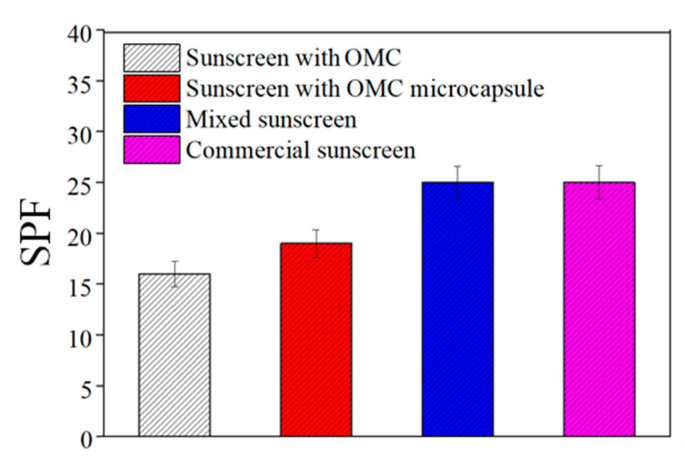
Comparison of SPF between microencapsulated sunscreen and various sunscreens.

The microcapsules were used in sunscreen and the SPF value of sunscreen was determined by an in vitro test [37]. The formula of sunscreen adopts the formula recommended in the 2015 edition of the safety technical specification for cosmetics [28]. The amount of OMC in the recommended formula is 3%, but the standard SPF value is 16.1. In this study, the sunscreen performance was compared with the recommended formula by adding the same amount of the OMC microcapsules into the sunscreen. In the same formula system, the SPF value of sunscreen used with OMC microcapsules was 19 (Figure 12).

**Figure 13:**
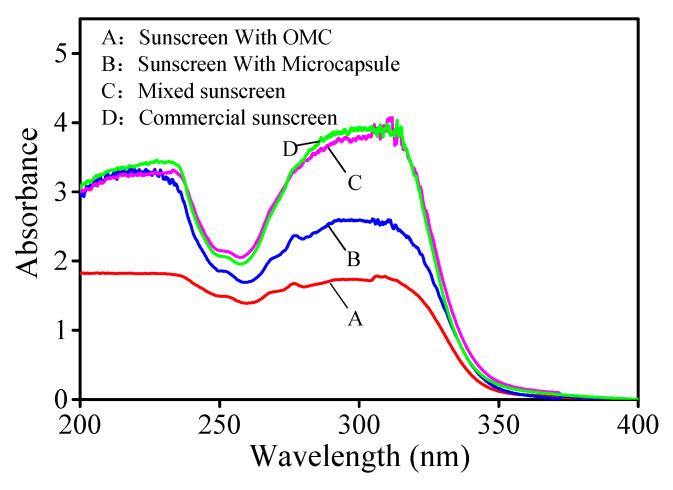
Ultraviolet absorption effect of sunscreen with the OMC microcapsule.

The addition into sunscreen of OMC was about 16%, which improved the sunscreen performance by 18.75%. In addition, the UV absorption of sunscreen containing the OMC microcapsules could be observed. As shown in Figure 13, the UV absorption effect of sunscreen containing OMC microcapsules is higher than that of sunscreen with OMC directly added [38]. In addition, this experiment also compared the microencapsulated sunscreen with the SPF value of 25 on the market. It was found that when the mass ratio of OMC microcapsule reached 6%, the SPF value of OMC microcapsule sunscreen was the same as that of sunscreen on the market. It was found that the UV absorption was enhanced only at 280–320 nm. This is consistent with the UV absorption effect of OMC which is a UVB-stage filter. It is proven that the enhancement effect of sunscreen is due to the increase of OMC. Through the application of sunscreen microcapsules in sunscreen products, it is found that the microencapsulated sunscreen agent can improve the sunscreen efficacy and can compete with the products on the market [39]. At the same SPF value, it can reduce the use of sunscreen, and has the effect of slow-release and reducing skin contact [40].

## 4. Conclusions

In this study, the OMC microcapsule was successfully prepared by complex coacervation based on SC and GA. The concentration of wall materials, the ratio of core materials and wall materials, and the amount of emulsifier can affect the shape and particle size of the microcapsules. The optimum preparation conditions of the OMC microcapsules were obtained as follows: wall core ratio 1:1, the curing agent content of 1%, pH = 2.5, 200 rpm, and encapsulation efficiency was 83.3%. The particle size of the capsules ranged from 5 to 16 μm, and the microcapsules were relatively uniform. The results of TG and FTIR indicated that OMC was successfully encapsulated by SC/GA. The SEM showed that the OMC microcapsule dispersed in spherical shape with micro-size. The release behaviors indicated that the OMC microcapsule could effectively inhibit OMC release and showed sustained release behavior compared to free OMC. The UV absorption suggested that the sunscreen effect of the OMC microcapsule was better than free OMC due to the ultraviolet absorption of natural polymer (SC) and the reflection of microcapsules’ core-shell. It was found that the sunscreen performance and SPF value of sunscreen containing OMC microcapsules were higher than those of sunscreen with the same amount of OMC. The sunscreen efficiency can be increased by 18.75% under the same SPF value. The sustained-release effect of sunscreen microcapsules can make the sunscreen performance more lasting. The shielding effect of microcapsules can reduce the direct contact between sunscreen and skin. The results indicated that the OMC microcapsules prepared by complex coacervation would have good potential to be used in sunscreen products.

## Figures and Tables

**Table 1:** Summary of experimental conditions.

	Stirring Speed	Ratio of Core Material to Wall Material	Curing Agent Content
Orthogonal	200	1:2	1%
	400	1:1	2%
	600	2:1	3%

**Table 2:** Sunscreen formula tested in the experiments.

Components	Mass Fraction (%)
Phase A	
Cetearyl Alcohol	2.21
PEG-40 Castor oil	0.63
Sodium Cetearyl Sulphate	0.32
Decyl Oleate	15.00
Ethylhexyl Methoxycinnamate	3.00
Butyl Methoxydibenzoylmethane	0.50
Propylparaben	0.10
Phase B	
Water	53.57
2-Phenyl-Benzimidazole-5-Sulphonic Acid	2.78
Sodium Hydroxide (45% solution)	0.90
Methylparaben	0.30
Disodium Ethylene Diamine Tetraacetic Acid (EDTA)	0.10
Phase C	
Water	20.00
Carbomer	0.30
Sodium Hydroxide (45% solution)	0.30

**Table 3:** Zeta-potential, size, and PDI of particles with different samples ratio of GA and SC.

Sample	Zeta (mV)	Size (μm)	PDI
S1	10.09 ± 0.484	15.32 ± 0.457	0.348 ± 0.006
S2	8.47 ± 0.216	14.45 ± 0.289	0.432 ± 0.007
S3	7.14 ± 0.269	13.28 ± 0.305	0.332 ± 0.006
S4	4.43 ± 0.154	12.52 ± 0.401	0.323 ± 0.006
S5	2.93 ± 0.087	11.32 ± 0.232	0.301 ± 0.004
S6	1.56 ± 0.048	10.01 ± 0.012	0.202 ± 0.002
S7	−0.19 ± 0.006	8.32 ± 0.175	0.322 ± 0.003
S8	−0.85 ± 0.026	7.56 ± 0.173	0.345 ± 0.002
S9	−2.18 ± 0.087	5.53 ± 0.143	0.366 ± 0.004

Experiment conditions: S1: GA/SC = 0.1, S2: GA/SC = 0.2, S3: GA/SC = 0.4, S4: GA/SC = 0.6, S5: GA/SC = 0.8, S6: GA/SC = 1, S7: GA/SC = 1.2, S8: GA/SC = 1.4, S9: GA/SC = 1.6.

**Table 4:** Factors and levels of the OMC microcapsules.

Run	Speed (rpm)	OMC to GA/SC Ratio (g/g)	Curing Agent Content (%)	*EE* (%)
1	200	1:1	1%	83.3
2	200	1:2	2%	32.7
3	200	2:1	3%	53.4
4	400	1:1	2%	21.3
5	400	1:2	3%	38.9
6	400	2:1	1%	54.2
7	600	1:1	3%	61.2
8	600	1:2	1%	63.6
9	600	2:1	2%	22.7
K1	0.387	0.349	0.670	
K2	0.252	0.321	0.256	
K3	0.288	0.256	0.145	
*R*	0.135	0.093	0.670	

**Table 5:** Kinetic models for the OMC microcapsule release.

Models	Parameters			
Zero-order	**OMC**		**The OMC Microcapsule**	
	*k*	0.05385	*k*	0.02945
	*R^2^*	0.5705	*R^2^*	0.8034
First-order	*k*	0.003610	*k*	0.00277
	*R^2^*	0.9705	*R^2^*	0.9771
Higuchi	*k*	0.6877	*k*	0.5318
	*R^2^*	0.8409	*R^2^*	0.8041
Rigter-Peppas	*k*	0.6712	*k*	0.7924
	*n*	0.4571	*n*	0.5472
	*R^2^*	0.9812	*R^2^*	0.9964

## Data Availability

The data presented in this study are available on request from the corresponding author.
